# Parallel Loss-of-Function at the *RPM1* Bacterial Resistance Locus in *Arabidopsis thaliana*

**DOI:** 10.3389/fpls.2012.00287

**Published:** 2012-12-26

**Authors:** Laura Rose, Susanna Atwell, Murray Grant, Eric B. Holub

**Affiliations:** ^1^Institute of Population Genetics, Heinrich-Heine UniversitätDüsseldorf, Germany; ^2^Molecular and Computational Biology, University of Southern CaliforniaLos Angeles, CA, USA; ^3^School of Biosciences, University of ExeterExeter, UK; ^4^School of Life Sciences, University of WarwickWellesbourne, UK

**Keywords:** parallel evolution, host-parasite coevolution, adaptation, loss-of-function, balancing selection

## Abstract

Dimorphism at the Resistance to *Pseudomonas syringae* pv. maculicola 1 (*RPM1*) locus is well documented in natural populations of *Arabidopsis thaliana* and has been portrayed as a long-term balanced polymorphism. The haplotype from resistant plants contains the *RPM1* gene, which enables these plants to recognize at least two structurally unrelated bacterial effector proteins (AvrB and AvrRpm1) from bacterial crop pathogens. A complete deletion of the *RPM1* coding sequence has been interpreted as a single event resulting in susceptibility in these individuals. Consequently, the ability to revert to resistance or for alternative *R*-gene specificities to evolve at this locus has also been lost in these individuals. Our survey of variation at the *RPM1* locus in a large species-wide sample of *A. thaliana* has revealed four new loss-of-function alleles that contain most of the intervening sequence of the RPM1 open reading frame. Multiple loss-of-function alleles may have originated due to the reported intrinsic cost to plants expressing the RPM1 protein. The frequency and geographic distribution of *rpm1* alleles observed in our survey indicate the parallel origin and maintenance of these loss-of-function mutations and reveal a more complex history of natural selection at this locus than previously thought.

## Introduction

Plants use two evolutionarily conserved mechanisms to induce defense responses against potential pathogens. The first mechanism is provided by plant receptor-like proteins in the plasma membrane that detect conserved microbe molecules outside the host cell, often referred to as pathogen associated molecular patterns (PAMPs). This PAMP-triggered immunity (PTI) is robust and encompasses recognition of a wide variety of molecules such as bacterial flagellin, heat shock proteins and peptidoglycans, and fungal chitin oligomers (reviewed in Schwessinger and Zipfel, 2008). The second mechanism is triggered by predominately cytoplasmic localized receptor-like proteins that enable detection of numerous microbial effector proteins, which have been released within the host cell and have specific functions which collectively suppress defense and/or re-configure host metabolism to nourish pathogen growth. The suite of pathogen effectors varies considerably among pathogens, with bacterial pathogens capable of delivering tens of effectors, whereas fungi and oomycetes are predicted to deliver hundreds of effectors. The plant membrane bound and cytosolic receptor-like proteins typically share a structurally conserved leucine-rich repeat (LRR) domain.

The bacterial resistance gene *Resistance to Pseudomonas syringae pv*. *maculicola* 1 (*RPM1*), encodes a classic cytoplasmic receptor-like protein that is characterized by an amino terminal coiled-coil domain, a central nucleotide binding (NB) site and a carboxyl terminal LRR domain. *RPM1* was one of the first plant resistance genes to be molecularly described from studies of natural variation in *Arabidopsis thaliana* (Grant et al., 1995). Moreover, RPM1 was the first example of a dual specificity *R*-protein, recognizing the sequence-unrelated AvrB and AvrRpm1 effector proteins derived from bacterial pathogens of soybean and *Brassica* species, respectively (Bisgrove et al., 1994). Actual dual specificity recognition is through effector-mediated modifications of *R*PM1-*in*teracting protein 4 (RIN4) in the presence of the bacterial type III effector proteins AvrRpm1 or AvrB (Mackey et al., 2002). Functional characterization of RPM1 and its interacting components have provided tremendous insight into underlying plant disease resistance mechanisms, and has also provided an important precedent for theoretical consideration of evolutionary and ecological genetics of plant innate defense (Stahl et al., 1999; Tian et al., 2003).

The *RPM1* gene was shown to have originated prior to split between the ancestors of the *Brassica* and *Arabidopsis* lineages because a single homologous copy was found at two of six homologous loci in *Brassica napus*, a cultivated polyploid relative of *A. thaliana* (Grant et al., 1998). Susceptibility to bacteria expressing *AvrRpm1* has previously only been observed as a complete deletion of the coding sequence, replaced by a much shorter fragment of unknown origin in susceptible *A. thaliana* plants, with similar but independent *RPM1-*deletions in two of the four *rpm1-*null loci of *B. napus* (Grant et al., 1998).

This evidence, combined with analysis of the divergence in the flanking sequence between the null and functional RPM1 haplotypes in *A. thaliana*, has led to the conclusion that this locus represents an example of a long-term balanced polymorphism (Stahl et al., 1999). A mechanistic explanation of the maintenance of this polymorphism was provided by evidence for a cost of *RPM1* in the absence of the pathogen (Tian et al., 2003). Notably, no evidence has been found to indicate that alternative functional alleles segregate at this locus.

Assuming a cost of *RPM1* resistance in the absence of the pathogen, we hypothesized that more intensive sampling of *A. thaliana* within a defined geographic range could reveal other loss-of-function alleles arising from independent mutation events, unless sufficient gene flow existed among populations for this single deletion null allele to spread throughout the species range.

To test this prediction, we first surveyed variation at the *RPM1* locus in a UK-wide diversity collection of 89 *A. thaliana* accessions. Surprisingly, specific recognition of AvrRpm1 occurred at a much lower frequency of ca. 37%, suggesting lower disease pressure in the UK than reported in a global sample (Stahl et al., 1999; Aranzana et al., 2005). Molecular analysis of the *RPM1* locus was undertaken in these accessions and in an expanded global sample of 92 accessions. This revealed four unique RPM1 protein variants that fail to confer disease resistance to bacteria expressing AvrRpm1 or AvrB effectors. Our data suggests a more complex evolutionary history at the *RPM1* locus than previously indicated, in which multiple loss-of-resistance alleles are maintained in addition to *RPM1*-resistance specificity.

## Results

### The majority of *AvrRpm1* susceptible accessions do not have the *rpm*1-null deletion

**Figure 1:**
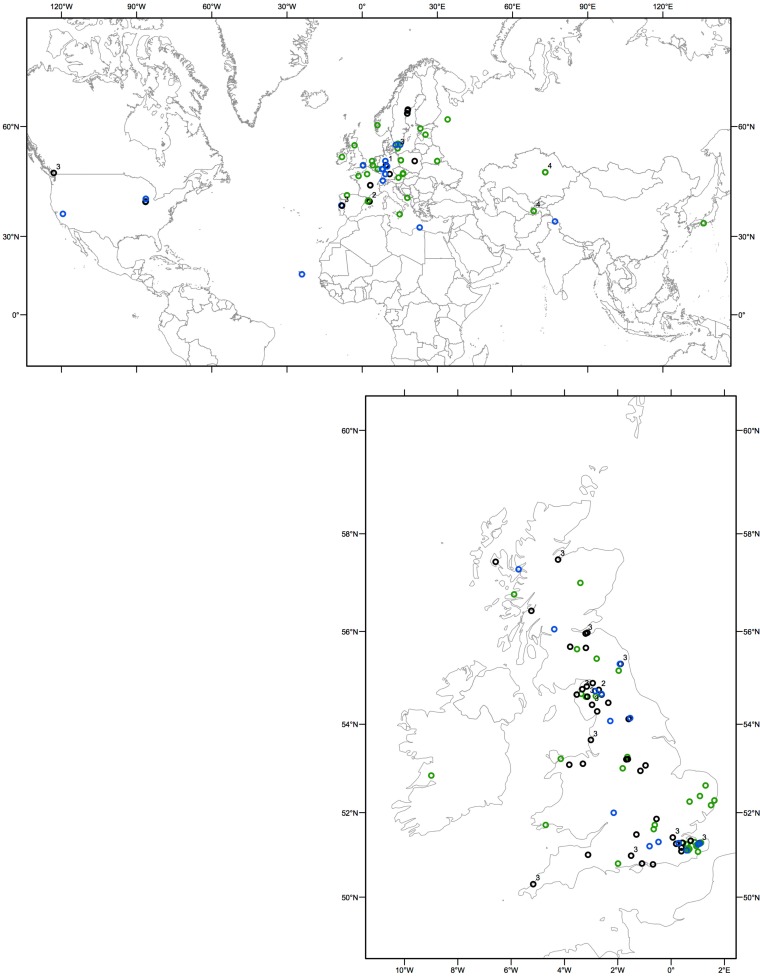
**Geographical distribution of resistant and susceptible individuals determined from HR and PCR**. Resistant accessions are represented by light green open circles, rpm1-deletion nulls by blue open circles. The susceptible accessions containing *RPM1* genes with premature stop codons are represented by black open circles. Next to these circles, the type of the loss-of-function allele (allele types 1 through 4 as defined in Figure 2; Table 1) is indicated.

Screening of a collection of 89 UK *A. thaliana* accessions for recognition of AvrRpm1 and AvrB by inoculation of the normally virulent *P. syringae pv. tomato* DC3000 (DC3000) modified to carry either *AvrRpm1* or *AvrB* revealed a significantly higher occurrence of *RPM1* compatible interactions (i.e., hosts were susceptible) than previously reported (Figure 1; Tables A1 and A2 in Appendix). In total, 63% of UK accessions were susceptible, based upon failure to restrict bacterial growth and absence of a visible macroscopic hypersensitive response. Examination of the susceptible accessions by PCR using primers specific for either the *RPM1* gene or the deletion null allele revealed that not all susceptible accessions had a deletion of *RPM1*.

In the UK sample, of the accessions that lacked resistance, only 25% contained the previously described deletion null allele (Figure 1; Table A1 in Appendix). Based on these unexpected observations, we expanded our study to include 92 global *A. thaliana* accessions. PCR based genotyping revealed that a larger proportion (43%) of the susceptible individuals from the global sample had the deletion null allele. This still represented <20% of all accessions in the combined UK and global samples (Figure 1; Tables A1 and A2 in Appendix).

A total of 38% of all accessions phenotyped were found to be susceptible yet contain the *RPM1* coding sequence (Figure 1; Tables A1 and A2 in Appendix). Our subsequent analysis of the entire *RPM1* coding region and more than 500 nucleotides upstream and 178 nucleotides downstream of *RPM1* in a sample of 31 *A. thaliana* accessions revealed loss-of-function (susceptibility), resulting from premature stop codons caused by single nucleotide substitutions or small indels (Table 1).

**Table 1:** **Protein sequence variation among *RPM1* alleles**.

	Position																			
Accession	99	106	116	149	150	151	215	304	375	403	534	541	623	664	698	703	779	Phenotype	Type	Cause
Col-0	W	R	Q	I	D	D	K	G	G	L	A	G	–	E	L	G	Q	R		
Ct-0	.	.	.	–	–	–	.	.	.	.	.	S	.	D	.	.	.	R		
Edi-1	.	.	.	–	–	–	.	.	.	.	.	S	.	D	.	.	.	R		
Bur-0	.	.	K	–	–	–	.	.	.	.	.	.	.	D	.	.	.	R		
Ard-1	.	.	K	–	–	–	.	.	.	.	.	.	.	D	.	.	.	R		
God-1	.	.	K	–	–	–	.	.	.	.	.	.	.	D	.	.	.	R		
Wim-1	.	.	K	–	–	–	.	.	.	.	.	.	.	D	.	.	.	R		
Ty-0	.	.	.	.	.	.	.	.	D	.	.	.	.	D	.	.	.	S		
Got-7	*	W	.	–	–	–	.	.	.	.	.	.	.	D	.	.	.	S	1	Non–sense mutation
Ts-5	.	.	.	–	–	–	.	.	.	.	.	.	A*	D	.	.	E	S	2	Single nucleotide insertion and non-sense mutation
Laz-1	.	.	.	–	–	–	.	.	.	.	.	.	A*	D	.	.	E	S	2	
Rrs-7	.	.	.	–	–	–	Q	.	.	.	.	.	.	D	.	.	.	R		
Lov-1	.	.	.	–	–	–	Q	.	.	.	.	.	.	D	.	.	.	R		
Amb-1	.	.	.	–	–	–	.	.	.	.	.	.	.	D	.	W	.	S		
Cnt-3	.	.	.	–	–	–	.	.	.	_	.	.	.	D	.	W	.	S	3	Non-sense mutation
Chs-1	.	.	.	–	–	–	.	.	.	_	.	.	.	D	.	W	.	S	3	
Asp-1	.	.	.	–	–	–	.	.	.	_	.	.	.	D	.	W	.	S	3	
Edi-2	.	.	.	–	–	–	.	.	.	_	.	.	.	D	.	W	.	S	3	
Hil-1	.	.	.	–	–	–	.	.	.	_	.	.	.	D	.	W	.	S	3	
Inv-1	.	.	.	–	–	–	.	.	.	_	.	.	.	D	.	W	.	S	3	
Ksk-2	.	.	.	–	–	–	.	.	.	_	.	.	.	D	.	W	.	S	3	
Mit-1	.	.	.	–	–	–	.	.	.	_	.	.	.	D	.	W	.	S	3	
Su-1	.	.	.	–	–	–	.	.	.	_	.	.	.	D	.	W	.	S	3	
Fei-0	.	.	.	–	–	–	.	.	.	_	.	.	.	D	.	W	.	S	3	
Van-0	.	.	.	–	–	–	.	.	.	_	.	.	.	D	.	W	.	S	3	
Cra-1	.	.	.	–	–	–	.	.	.	_	.	.	.	D	.	.	.	S	3	
Var2-1	.	.	.	.	.	.	.	V	.	_	.	.	.	D	.	W	.	S	3	
Roy-1	.	.	.	–	–	–	.	.	.	.	.	.	.	D	.	.	.	R		
Bra-1	.	.	.	.	.	.	.	.	.	.	.	.	.	D	I	.	.	R		
Ksk-1	.	.	.	.	.	.	.	.	.	.	.	.	.	D	I	.	.	R		
Kz-1	.	.	.	–	–	–	.	.	.	.	//*	–	.	D	.	.	.	S	4	Complex inversion, followed by a non-sense mutation
Kondara	.	.	.	–	–	–	.	.	.	.	//*	–	.	D	.	.	.	S	4	
				1st CAPs marker												2nd CAPs marker				

**Figure 2:**
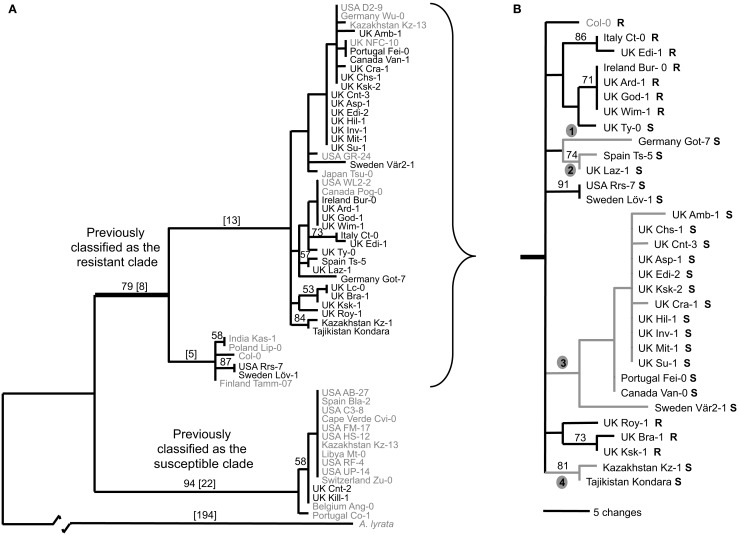
**Maximum parsimony analysis of *RPM1* region from a worldwide collection of *A. thaliana* indicating recurrent loss-of-function at the *RPM1* locus**. **(A)** Analysis based on the *RPM1* coding region (2781 nucleotides), 2190 nucleotides upstream, and 176 nucleotides downstream of coding region. **(B)** Analysis based on coding region and 500 nucleotides upstream and 176 nucleotides downstream of coding region. Allele sequences new in this study are black; sequences from Stahl et al. (1999) are gray. No sequence data was available for the accessions from Stahl et al. from 1743 bp onward. These positions were treated as missing data in the analysis. Bootstrap values are indicated above branches; branch lengths are in square brackets. Resistance (R) or susceptibility (S) based on HR to DC3000 carrying *AvrRpm1* indicated next to accession name. The four new branches on which loss-of-function substitutions occurred are indicated. The specific substitutions are found in Table 1.

*Position indicates the predicted amino acid position using Col-0 as a reference. Invariant positions across alleles are removed. Dots indicate positions matching the Col-0 reference allele, while a hyphen indicates a gap. Stars indicate stop codons. The two CAPs markers corresponding to polymorphic sites 149 to 151 and site 703, respectively, are indicated. The phenotypic response upon infection with DC3000 with *AvrRpm1* for each accession is reported as Resistant (R) or Susceptible (S). The type of loss-of-function mutations (type 1 through 4) corresponding to the branches on the phylogeny (Figure 2) and the cause for the loss-of-function is located in the far right columns*.

Four novel independent loss-of-function alleles were identified (Figure 2B; Table 1) and their occurrence varied in frequency in our sample (Table 1). At one extreme, a single accession from Germany had a premature stop codon at amino acid position 99. At the other extreme, 13 susceptible individuals shared a premature stop codon at amino acid position 403. These 13 individuals included accessions from both Europe and North America. The other two loss-of-function mutations were each found in a pair of accessions. One allelic type contains a simple frameshift mutation that leads to a premature stop codon at amino acid position 625. This mutation is found in one accession from the UK and one accession from Spain.

A more complicated mutational scenario underlies the loss-of-function mutation found in the accessions from Kazakhstan and Tajikistan. The *RPM1* alleles from these two individuals encode a premature codon at amino acid position 509. This stop codon is embedded in a large insertion of 225 nucleotides at this part of the *RPM1* gene. At the site of the insertion, these two alleles are missing 130 nucleotides of the *RPM1* gene relative to the other alleles. However, the 225 bp insert contains these missing 130 nucleotides in reverse complement form. In fact the entire 225 insert is a 100% match to the reverse complement of this region of the other *RPM1* alleles sampled, and includes the missing 130 nucleotides plus an additional 12 bp from the left border and 83 nucleotides matching the right border of the indel position. In contrast to the other loss-of-function mutations that involve single nucleotide substitutions, this loss-of-function allele appears to be derived from a complex mutational event, possibly via a double strand break and repair mechanism.

### The loss-of-function alleles arose independently and are younger than the deletion null allele

Orthologs of *RPM1* are present in close relatives of *A. thaliana*, indicating that the ancestral allele at this locus was most likely a functional version of *RPM1*. The significant haplotype divergence between the deletion null and *RPM1* containing haplotypes indicates that the deletion null predates the other loss-of-function alleles. Phylogenetic analysis of the flanking region and the *RPM1* coding sequence revealed that the novel loss-of-function alleles cluster within the clade previously described to be the “resistance haplotype” (Figure 2). This implies that these loss-of-function mutations are derived from previously functional alleles. Furthermore, each loss-of-function represents a unique mutation and these losses are predicted to have occurred independently.

**Figure A1:**
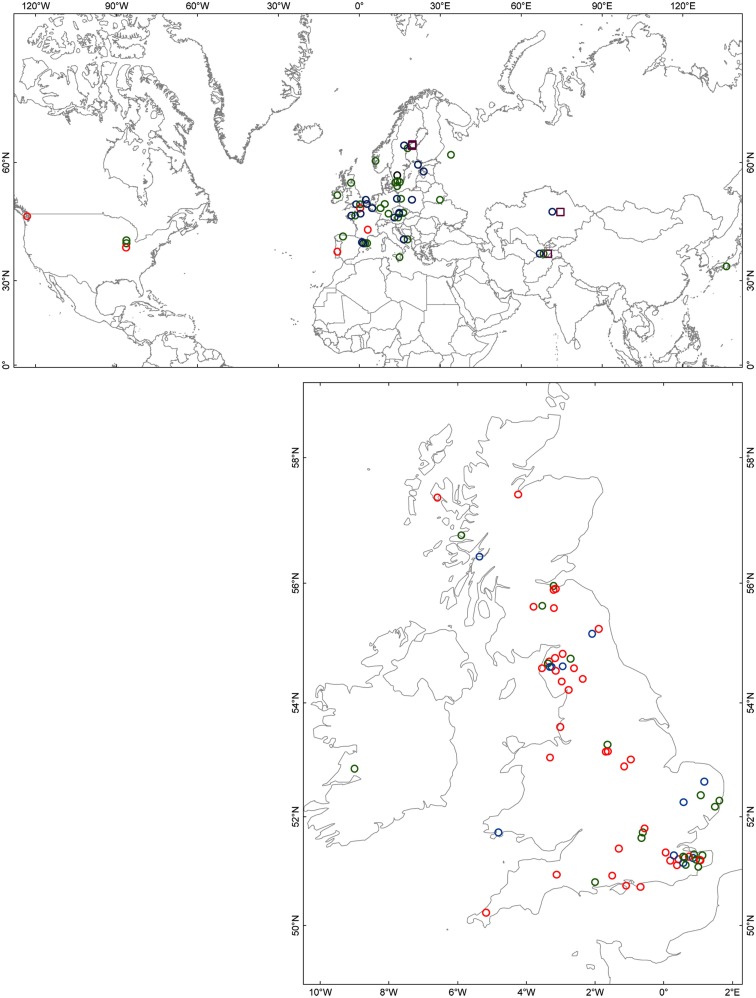
**Geographical distribution of phenotyped accessions in which genotypes have been defined based on the diagnostic CAPs markers**. Accessions with the I^149^D^150^D^151^ deletion (CAPs marker 1) and the G^703^ > W^703^ (CAPs marker 2) are represented by open red circles; presence of only CAPs marker 1 by open green circles; presence of only CAPs marker 2 by a black open circle; matching Col-0 reference (i.e., lacking both diagnostic markers) by blue open circles; presence of the larger indel/inversion as found in Kz-1 and Kondara by open purple squares. (This indel/inversion was detected by the digestion pattern of CAPs marker 2 in combination with a larger PCR fragment size).

To survey the sequence diversity in our larger collection of plants, we developed two diagnostic Cleaved Amplified Polymorphic Sequences (CAPs) markers (Table A3 in Appendix). The presence of either marker alone was not associated with susceptibility, however all individuals carrying both CAPs markers (44 out of 172 screened individuals) were susceptible. This combination of markers was found in nearly all individuals of clade 3 of the *RPM1* gene tree (Figure 2B) and this loss-of-function allele appears to be widespread in its distribution (Figure A1 in Appendix).

### Haplotype diversity around the *RPM1-*deletion junction

**Figure 3:**
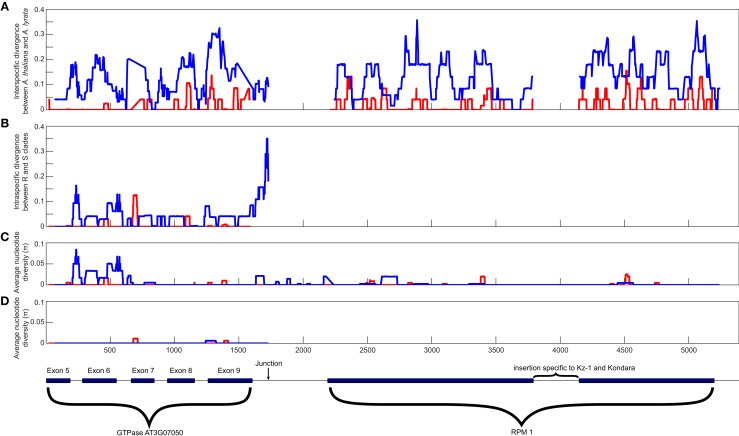
**Sliding window analyses across the RPM1 and NSN1 region**. **(A)** Interspecific divergence between *A. thaliana* and *A. lyrata*. **(B)** Intraspecific divergence between *A. thaliana* alleles belonging to the R and S clades. **(C)** Intraspecific polymorphism among alleles carrying the *RPM1* gene. **(D)** Intraspecific polymorphism among alleles carrying the *RPM1-*deletion null. Silent polymorphisms include both synonymous polymorphisms and polymorphisms in non-coding regions, such as introns. The structure of the genes is shown below the graph. Boxes indicate exons, solid lines indicate introns. Sliding window analyses were conducted using DnaSP v. 5 (Librado and Rozas, 2009). Values are midpoints of 25 bp windows.

The haplotype differentiation, which is visible on the phylogenetic tree and in the sliding window analysis (Figures 2 and 3), results in statistically significant positive values of Tajima’s D within the flanking regions (Table 2). This marked haplotype differentiation at the deletion junction has been reported in previous analyses and was interpreted as a hallmark of balancing selection (Stahl et al., 1999). Upon combined examination of the previously reported sequences and of our new sequences, we observed that a substantial portion (92%) of the previously analyzed region upstream of *RPM1* contains a region predicted to encode the *NSN1* gene (Figure 3). The coding region of this gene shows evidence of evolutionary constraint with π_non_/π_syn_ = 0.181 (within *A. thaliana*) and Ka/Ks = 0.15 (divergence to *A. lyrata*). However, immediately downstream of the *NSN1* gene, silent polymorphism within *A. thaliana* shows a sharp increase (π_sil_ in coding region = 0.02015 < π_sil_ downstream of *NSN1* = 0.07622). Furthermore, of the 21 fixed differences between the two *A. thaliana* haplotypes, 11 reside in the short 137 bp interval downstream of the stop codon, while the other 10 are distributed across the 1600 nucleotide coding region of the *NSN1* gene. Models of long-term balancing selection would predict a gradual increase in allelic divergence over the entire interval, yet these data show divergence is 12 times higher immediately downstream of the stop codon of the *NSN1*, compared to within the *NSN1* gene.

While this peak in divergence between haplotypes in *A. thaliana* in the vicinity of the deletion junction has previously been interpreted as evidence of a long-term balanced polymorphism, new information about the gene content in this segment of the *A. thaliana* genome reveals that this marked peak may be partially related to differences in the form and strength of natural selection operating in parallel on the *NSN1* gene. Haplotype differentiation between the resistant haplotype and deletion null haplotype extends across the *NSN1* coding region and the jump of divergence between haplotypes occurs specifically in the non-coding region between these genes. Combined with the observation of multiple, independent loss-of-function mutations at the *RPM1* locus in *A. thaliana*, our data suggest a much more dynamic situation exists at the *RPM1* disease resistance locus than previously proposed and we can reject strict bi-allelic balanced polymorphism at *RPM1*.

### Protein sequence variation within RPM1 coding region

Previous studies of this locus focused primarily on the sequence evolution flanking the *RPM1-*deletion junction and did not address sequence variation within *RPM1*. Based on analyses of an interspecific pair of *RPM1* alleles, Stahl et al. state that the “selectively driven turnover of resistance alleles does not appear to be important in RPM1 protein evolution.” However, in our analyses of 31 *RPM1* alleles (plus the reference Col-0 sequence) we observe a significant excess of amino acid polymorphism relative to the neutral expectation in the *RPM1* alleles (McDonald–Kreitman test, *p*-value = 0.028, Table 2). Elevated amino acid polymorphism within the *RPM1* coding region cannot be attributed exclusively to the presence of loss-of-function alleles, because functional and non-functional alleles contribute nearly equal numbers of non-synonymous segregating sites (five versus six respectively), although there are nearly 1.5 times as many non-functional alleles in our sample (Table 2). The maintenance of multiple amino acid variants of both functional and non-functional alleles is consistent with a history of diversifying selection, similar to that reported at *Pto* in wild tomatoes (Rose et al., 2005, 2007).

**Table 2:** **Summary statistics and tests of neutrality**.

Data set	Sampled individuals	Included gene regions	Position in alignment	Sites without gaps	Haplotypes	π^a^ (non, syn, sil)	π_non_/π_syn_	S^b^ (non, syn)	Divergence to *A. lyrata*	Min. No. Rec^c^	TD (*p*-value)^d^	MK *p*-value^e^
Stahl et al. sequences	28	NSN1 + intergenic	1–1741	1663	12	0.0129 (0.003, 0.0166, 0.0265)	0.181	49 (8,11)	0.054	0	2.66 (*p* < 0.01)	*p* = 0.489
Stahl et al. plus new sequences	48	NSN1 + intergenic	1–1741	1006	14	0.0102 (0.003, 0.0107, 0.0214)	0.28	33 (8,6)	0.059	1	1.27 (NS)	*p* = 0.624
Longest available sequences	23	intergenic + RPM1	662–5376	4303	18	0.00125 (0.00082, 0.00117, 0.00195)	0.701	31 (13,6)	0.047	6	−1.37 (NS)	*p* = 0.0331
Largest inclusive set of RPM1 sequences	32	intergenic + RPM1	1686–5376	3320	18	0.00129 (0.00092, 0.00137, 0.00189)	0.672	26 (11,5)	0.042	4	−1.19 (NS)	*p* = 0.0678
RPM1 coding only	32	RPM1	2191–5198	2642	14	0.00121 (0.00093, 0.00137, 0.00181)	0.679	16 (11,5)	0.042	2	−1.07 (NS)	*p* = 0.0280
Functional alleles only	12	RPM1	2191–5198	2772	6	0.00073 (0.00072, 0.00076, 0.00108)	0.947	7 (5,2)	0.042	0	−0.49 (NS)	*p* = 0.112
Non-functional alleles only	18	RPM1	2191–5198	1642	7	0.00081 (0.00070, 0.00118, 0.00118)	0.593	9 (6,3)	0.042	1	−0.65 (NS)	*p* = 0.153

*^a^ Average pairwise differences*.

*^b^ Segregating sites*.

*^c^ Minimum number of recombination events based on four gamete test (Hudson and Kaplan, 1985)*.

*^d^ Tajima’s *D* statistic and *p*-value (Tajima, 1989); all sites analyzed*.

*^e^*p*-value based on McDonald–Kreitman test (McDonald and Kreitman, 1991)*.

## Discussion

Evidence for parallel loss-of-resistance and maintenance of multiple mutant alleles from this study provides a new dimension to our understanding of how natural selection shapes genetic variation of resistance genes in plants. Although loss-of-function mutations are not uncommon for traits that are no longer required in an organism (for example eyesight in cave dwelling animals), it is surprising to observe the maintenance of multiple loss-of-function alleles at a locus that is expected to help protect the organism against disease.

An intriguing question highlighted in the original RPM1 study was the discrepancy between the levels of within haplotype diversity and the actual observed frequency of the two haplotypes within *A. thaliana*. The deletion null haplotype was sampled at a frequency of 0.48, although it harbored 10-fold less polymorphism than the resistant haplotype at a frequency of 0.52. Furthermore, since the branches separating these two haplotypes carried nearly equivalent numbers of derived polymorphisms relative to an outgroup, the polymorphism appeared to be stable over a long period of time and the alleles were assumed to be nearly the same age. Theoretical modeling revealed that cyclical fluctuations of the two haplotypes over a very long period of time could account for these observations, although the mean effective population size of the resistance allele in these models was required to exceed the sampled frequency to fit the data. With the acquisition of additional data, we now know that multiple loss-of-function alleles segregate within *A. thaliana* and therefore, the long-term effective population size of a single loss-of-function class may be indeed accurately reflected in the level of within haplotype diversity. If multiple, alternative loss-of-function alleles segregate in the species, then theoretical models do not need to account for this extreme discrepancy in within class polymorphism.

Based on analysis of the flanking sequence of these alleles, we have established that the additional loss-of-function alleles are younger than the RPM1-deletion null first reported, and are independently derived from individuals carrying the full-length RPM1 allele. Segregation of multiple alleles that appear to be selectively equivalent is one hallmark of soft selective sweeps, in which independent (putatively selectively advantageous) mutations increase in frequency simultaneously. Soft sweeps are likely when the effective population size is large or the allelic mutation rate is high. In particular, when the adaptive allele involves a loss-of-function, a high proportion of these adaptations may result from recurrent mutation (Pennings and Hermisson, 2006). This may specifically apply in the case of RPM1 loss-of-function mutations.

A further question is whether to view these loss-of-function alleles as advantageous (since these individuals are consequently no longer able to recognize certain pathogen strains) or whether this is simply an example of relaxed selective constraint. Since the RPM1 gene is reported to carry a fitness cost in the absence of pathogen infection (Tian et al., 2003), it may be possible to consider the loss of RPM1 resistance as a specific adaptation to local environmental conditions. Since pathogen populations are notoriously variable in time and space, natural selection may have played a role in driving the loss of the RPM1 function in the absence of pathogen pressure. The limited outcrossing in *A. thaliana* may also restrict the spread of alleles throughout the species’ range. This would explain why the original RPM1-deletion allele is not the only “solution” to the fitness cost in the absence of pathogen pressure.

Another consideration is that alternative loss-of-function alleles may not be functionally equivalent, especially in the face of recurrent changes in the adaptive landscape typical of host-parasite interactions. Clearly, a complete gene deletion does not have the same probability of reverting to a functional allele as an allele with a single or few nucleotide changes. After a gene’s function has been altered (perhaps through a missense, non-sense, or frameshift mutation), it may serve as a repository of genetic variation for future bouts of selection. Gene disruption by relatively simple nucleotide changes may be only transient, while a complete deletion is typically not reversible.

This interpretation is in line with the “less is more” hypothesis of Olson (1999). He hypothesized that simple loss-of-function alleles may represent a reservoir of “near-functional” alleles and if the adaptive landscape is subject to frequent changes, reversion of previously disrupted alleles will be a common means of adaptation. He envisioned this hypothesis originally to explain examples of adaptive loss-of-function that apparently spread rapidly through early human populations. However, it now appears to be widespread, as cases for multiple, independent loss-of-function alleles have been shown to occur extensively throughout the microbial, animal, and plant kingdoms (Olson, 1999; Johanson et al., 2000; Protas et al., 2006; Kivimaki et al., 2007; Jeong et al., 2008; Ordonez et al., 2008; Dworkin and Jones, 2009; Kingsley et al., 2009; Kovach et al., 2009; Stergiopoulos and de Wit, 2009).

Indeed, the “less is more” proposal provides a more accurate description of evolution at the *RPM1* locus than previously reported. Unlike the original null allele described at the *RPM1* locus that resulted from a complete deletion of the coding sequence, the novel alleles we discovered are more recent in origin and have independently arisen from functional *RPM1* alleles. An intrinsic cost of expressing the RPM1-resistance protein in the absence of selection pressure by the corresponding pathogen may have been an important factor allowing the persistence of these independent mutant alleles in wild *A. thaliana* populations (Tian et al., 2003).

An exemplification of the adaptive potential of a single copy *R*-gene in *A. thaliana* is the downy mildew resistance gene *Recognition of Peronospora Parasitica 13* (*RPP13*; Rose et al., 2004; Hall et al., 2009). Multiple, functionally differentiated alleles are maintained at *RPP13* and this diversity is matched by allelic variation in the corresponding proteins of an oomycete thought to be an endemic parasite in European populations of *A. thaliana* (Allen et al., 2004; Holub, 2008; Hall et al., 2009). Likewise, multiple protein variants (both functional and non-functional) segregate at *RPM1*. However, protein polymorphism is not reported in the two bacterial proteins that are known to interact with RPM1 (i.e., neither *AvrB* or *AvrRpm1* show striking patterns of diversification across pathovars of *P. syringae*; Bisgrove et al., 1994). Instead, these bacterial proteins appear pathologically dispensable under laboratory conditions and can be readily excised from the pathogen. Therefore, the diversification in RPM1 protein sequence may not be directly driven by diversifying selection imposed by coevolution with these two pathogen proteins. Instead the selection may be on RPM1’s ability to recognize and respond to changes elicited by effectors such as AvrRpm1 and AvrB to RIN4 (Chung et al., 2011). Thus, RPM1 may have additional recognition specificities that have not been revealed through the narrow effector repertoire on which it has been tested to date. Alternatively, RPM1 may contribute other fitness benefits: R proteins are emerging as important components of the integrated stress response, and what we consider to be *rpm1* loss-of-function mutations may in fact confer a selective advantage in other environments (Chini et al., 2004).

Our data supports previous reports that *RPM1* resistance is an ancestral trait in the Brassicaceae. However, the frequency and geographic distribution of the multiple *rpm1* alleles indicate the parallel origin and persistence of loss-of-function mutations at this locus in *A. thaliana*. Further empirical evidence is required to determine whether the novel alleles can in fact be maintained without positing a microbial selection pressure. In this respect, the expanding assortment of *rpm1* alleles highlights the importance of this locus as an example for ecological genetics and the need for field experiments to compare the relative intrinsic costs of the new *rpm1* alleles.

## Materials and Methods

### Plant materials and disease resistance phenotyping

The seed collection of *A. thaliana* accessions from the UK was assembled by E. Holub. Seeds from the global sample were obtained from the Nordberg collection (Aranzana et al., 2005; available from the Arabidopsis stock centers). Five-week old rosettes were phenotyped for *RPM1* disease resistance using abaxial leaf inoculation of DC3000 modified to carry *AvrRpm1* or *AvrB* (2 × 10^8^ cfu ml^−1^) via needle-less syringe. Resistance (hypersensitive response, HR), evident as rapid collapse of the infiltrated leaf, was recorded after 6 h and susceptibility (no evident leaf collapse) at 20 h post inoculation. Response to carrier isolate (DC3000) was also determined after 6 h and 20 h to ensure that the phenotype was due to *AvrRpm1* interaction and not recognition of effectors delivered by the carrier (none were observed). Response to *AvrB* was identical to *AvrRpm1* and as such is not mentioned further.

*In planta* growth analysis was carried out using the same inoculation technique but at a reduced bacterial concentration (2 × 10^6^ cfu ml^−1^). Three days post inoculation bacterial multiplication was determined as follows: Three leaf disks per plant were excised, homogenized in 10 mM MgCl^2^, and the suspension serially diluted. Diluted leaf disk homogenates were plated on King’s Broth (King et al., 1954) containing rifampicin (50 μg/ml; DC3000) or rifampicin and kanamycin (50 μg/ml; DC3000*AvrRpm1* or DC3000*AvrB*), incubated for 36 h at 28°C, before colonies were counted. Resistant accessions had at least a 10-fold difference in bacterial growth between DC3000 carrying *AvrRpm1* and the virulent DC3000 strain.

### PCR amplification and sequencing

Alleles of the *RPM1* region were amplified by PCR using Taq (Promega, Madison WI). The *RPM1* locus was amplified in all accessions from 194 bp upstream of the start codon to 697 bp downstream of the termination codon. *RPM1* specific primers were designed to amplify this region in seven overlapping products (Table 3). To identify accessions containing the *rpm1*-deletion null, primers located 1700 bp upstream and 990 bp downstream from the *RPM1-*deletion junction were used. Expected amplicon length was 2733 bp in accessions lacking the *RPM1* gene and 5513 bp in accessions with the full-length *RPM1* gene. The upstream region of the *RPM1* gene was also amplified and sequenced. This region contains the 3′ half of the *NSN1* gene and a non-coding region directly upstream of *RPM1*. This region was amplified using two sets of primer pairs.

**Table 3:** **Sequences and annealing positions of primers used in this study**.

Target	Position	Primer 1	Primer 2
RPM1 (upstream)	697 bp upstream of start codon to 129 bp downstream of start codon	GATCTGATGGACGGAGATTATG	GGACTTCATGATCAGCAACTC
RPM1 (coding)	4 bp upstream of start codon to 642 bp downstream of start codon	GAAGATGGCTTCGGCTACTG	GAAGATATTCGCTGAGAGTGTAG
RPM1 (coding)	543 bp downstream of start codon to 1170 bp downstream of start codon	GCTCATCGGACGGCTTCTAAG	CTTGGTTGACATCATGCTTCC
RPM1 (coding)	1073 bp downstream of start codon to 1676 bp downstream of start codon	GAACGCAGAATTTGGAGCCGATAG	GTTGCACGTATACTATCAGGTG
RPM1 (coding)	1576 bp downstream of start codon to 2184 bp downstream of start codon	GATGACAGTGATGGTGATGATG	CAGTGAATCGCACAAGTCTCTTC
RPM1 (coding)	2068 bp downstream of start codon to 9 bp downstream of stop codon	GACTGCTTCAACGCAGAAGATG	CTTGGCCGCCTAAGATGAGAG
RPM1 (coding)	position 2585 bp downstream of start codon to 194 bp downstream of stop codon	GGTTAGAGTACGTACCAAGAG	CACTGACTCATGAGACCAGC
NSN1 (coding)	1828 bp downstream of start codon to 7 bp downstream of stop codon	TGAAGGAAATTCTCAAGCTTTGTCC	CAGACCGAGTCATTTACAAG
NSN1 (coding and intergenic)	2369 bp downstream of start codon to 254 bp downstream of stop codon	GAGTGATTGGAAACCACAACG	CGGGTGTTGTTTTCGTCATTCG
*rpm1*-deletion junction	1700 bp upstream of deletion junction to 990 bp downstream of deletion junction	GTCCTGGAGTTGTGATGTTG	GCGACTCTCTGGTATCTATC

Pooled PCR samples (four reactions per primer combination) from the seven *RPM1* specific products and the *NSN1* gene and 5′ *RPM1* region from each of the 31 accessions were gel purified on 1.5% agarose gel and subsequently purified using QIA quick PCR Purification Kit (Qiagen). PCR products were directly sequenced on an ABI3700 capillary sequencer (Applied Biosystems). Sequence traces were aligned to the original *RPM1* sequence obtained from Col-0 and base-calling was completed manually (Grant et al., 1995; NCBI Gene ID: 819889). All sequences are available from GenBank (Accession numbers: KC211311–KC211321 and KC249727–KC249748).

### Sequence analysis

Phylogenetic analysis, implemented in PAUP 4.0 beta 10, was used to infer the likely ancestry of the novel *rpm1* loss-of-function alleles (Swofford, 1999). The phylogenetic relationships between these sequences were inferred using maximum parsimony and neighbor joining (using the HKY85 model of substitution). These methods yielded similar topologies. Two datasets were analyzed. The first consisted of the *RPM1* coding region (2781 nucleotides), 2190 nucleotides upstream, and 176 nucleotides downstream of coding region. The second dataset was based on the *RPM1* coding region and 500 nucleotides upstream and 176 nucleotides downstream of coding region. No sequence data was available for the accessions from Stahl et al. (1999) from 1743 bp onward. These positions were treated as missing data in the analysis. The standard summary statistics, tests of neutrality, and sliding window analyses were performed using DnaSP v. 5 (Librado and Rozas, 2009). For the sliding window analysis, window size was set to 25 sites and step size was set to 1.

## Conflict of Interest Statement

The authors declare that the research was conducted in the absence of any commercial or financial relationships that could be construed as a potential conflict of interest.

## Appendix

**Table A1:** ***RPM1* genotypes and resistance phenotypes across UK accessions**.

Accession	Origin	Genotype (determined by PCR amplification)	*HR* to *AvrRpml*	*In planta* growth analysis
Aco-1	Acorn bank	*RPM1*	X	S
Amb-1	Ambleside	*RPM1*	X	S
Asp-1	Aspatria	*RPM1*	X	S
Ban-2	Bangor	*RPM1*	√	na
Bea-1	Bearsted	*RPM1*	√	R
Bek-1	Bekonscot	*RPM1*	√	R
Bid-1	Biddenden	*RPM1*	√	R
Big-1	Biggar	*RPM1*	√	R
Bra-1	Braithwaite	*RPM1*	X	R
Brm-1	Braemar	*RPM1*	X	na
Bur-0	Burren	*RPM1*	√	R
Cal-2	Calver	*RPM1*	√	R
Cam-1	Cambo	*RPM1*	√	R
Chi-1	Chilham	*RPM1*	√	R
Chr-1	Chartham	*rpml-null* (deletion)	X	na
Chs-1	Chiselhurst	*RPM1*	X	S
Cnt-1	Canterbury	*RPM1*	X	S
Cnt-2	Canterbury	*rpml-null* (deletion)	X	S
Cnt-3	Canterbury	*RPM1*	X	S
Coc-1	Cockermouth	*RPM1*	√	R
Cra-1	Cragside	*RPM1*	X	S
Crl-1	Carlisle	*RPM1*	X	S
Cul-1	Culgaith	*rpml-null* (deletion)	X	S
Duc-1	Dunvagen	*RPM1*	X	S
Dun-1	Dunwich	*RPM1*	√	R
Edi-1	Edinburgh	*RPM1*	√	R
Edi-2	Edinburgh	*RPM1*	X	S
Ema-1	East Mailing	*RPM1*	X	S
Fab-1	Fountains Ab.	*RPM1*	X	na
Far-1	Farnham	*rpml-null* (deletion)	X	na
Fav-1	Faversham	*RPM1*	√	R
Frd-1	Fordwich	*RPM1*	√	R
God-1	Godmersham	*RPM1*	X	R
Haw-1	Hawich	*RPM1*	√	na
Hil-1	Hilliers Arboretum	*rpml-null* (deletion)	X	S
Igt-1	Igtham	*RPMl-null*	X	S
Inv-1	Inverness	*RPM1*	X	S
Kil-1	Killearn	*rpml-null* (deletion)	X	S
Ksk-1	Keswick	*RPM1*	√	R
Ksk-2	Keswick	*RPM1*	X	S
Kyl-1	Kyle of Localsh	*rpml-null* (deletion)	X	S
Lan-1	Lanark	*RPM1*	X	S
Laz-1	Lazonby	*RPM1*	X	S
Leg-1	Legburthwaite	*RPM1*	X	S
Lha-1	Lower Harbledown	*RPM1*	X	S
Lwe-0	Little Welnetham	*RPM1*	√	R
Mit-1	Mithian St.	*RPM1*	X	S
Nor-1	Norwich	*RPM1*	√	R
Not-1	Nottingham	*RPM1*	X	S
Nun-1	Nunnery walk	*RPM1*	X	S
Pdw-1	Paddock wood	*RPM1*	X	S
Pee-1	Peebles	*RPM1*	X	S
Ply-1	Penrith	*RPM1*	√	R
Poo-1	Pooley bridge	*RPM1*	√	R
Rip-1	Ripon	*rpml-null* (deletion)	X	S
Rot-1	Rothbury	*rpml-null* (deletion)	X	na
Sco-1	Scotney castle	*RPM1*	√	S
Set-1	Settle	*rpml-null* (deletion)	X	na
Sev-1	Sevenoaks	*RPM1*	X	S
Sis-1	Sissinghurst	*rpml-null* (deletion)	X	S
Sit-1	Sittingbourne	*RPM1*	X	S
Sta-0	Stanton-dy Da	*RPM1*	√	na
Sma-1	Smarden	*RPM1*	√	R
Sna-1	Snape malting	*RPM1*	√	R
Som-1	Sommerset	*RPM1*	X	S
Sou-1	Southwell	*RPM1*	X	S
Siz-1	Sizergh castle	*RPM1*	X	S
Su-1	Southport	*RPM1*	X	S
Tea-1	Tewksbury	*rpml-null* (deletion)	X	S
Ty-0	Taynuilt	*RPM1*	X	S
Unt-1	Unthank	*rpml-null* (deletion)	X	S
Wen-1	Worlds end	*RPM1*	X	S
Wig-1	Wigton	*RPM1*	X	S
Wim	Wimborne	*RPM1*	√	R
Wis-1	Wisley	*rpml-null* (deletion)	X	S
Wma-1	West mailing	*RPM1*	√	R
Bog2	Bognor regis	*RPM1*	X	S
Lee-1	Leeds castle	*RPM1*	√	R
Bet-1	Betws-y-coed	*RPM1*	X	na
Bak-0	Bakewell	*RPM1*	X	S
Roy-1	Roydon	*RPM1*	√	R
Che-1	Chesham	*RPM1*	√	R
Rut-1	Ruthin	*RPM1*	X	S
Fof-1	Firth of Forth	*RPM1*	X	S
Sau-0	Saundersfoot	*RPM1*	√	R
Por	Portsmouth	*RPM1*	X	S
Chat-0	Chatsworth	*RPM1*	X	S
Whiz-1	Whipsnade	*RPM1*	X	S
Ard-1	Artoe	*RPM1*	X	R

**RPM1* genotypes were defined based on presence or absence of *RPM1* determined by PCR amplification using *RPM1* specific primers, with *rpm1*-null lacking entire *RPM1* sequence. Resistance was assessed using two independent assays: (A) Presence of hypersensitive response (HR) following inoculation of DC3000 specifically expressing *AvrRpm1*, but not following inoculation of DC3000 with empty vector; (B) Specific inhibition of bacterial growth *in planta* following inoculation of DC3000 expressing *AvrRpm1* (see Materials and Methods). The accessions Bra-1, God-1, and Ard-1 were determined to be resistant although they failed to elicit an HR upon inoculation with DC3000 specifically expressing *AvrRpm1**.

**Table A2:** ***RPM1* genotypes and resistance phenotypes across world accessions**.

Accession	REGION	Genotype (determined by PCR amplification)	*HR* to *AvrRpml*
An-1	Belgium	RPM1	√
Bor-1	Czech Republic	*RPM1*	√
Bor-4	Czech Republic	*RPM1*	√
Lp2-2	Czech Republic	*RPM1*	√
Lp2-6	Czech Republic	*RPM1*	√
Zdr-1	Czech Republic	*RPM1*	√
Zdr-6	Czech Republic	*RPM1*	X
Uod-1	Austria	*RPM1*	√
Uod-7	Austria	*RPM1*	√
Van-0	Canada	*RPM1*	X
Cvi-0	Cape Verde Isl	*rpml-null* (deletion)	X
Pu2-23	Croatia	*RPM1*	√
Pu2-7	Croatia	*RPM1*	√
Br-0	Czech Republic	*RPM1*	√
Cibc-17	England	*rpml-null* (deletion)	X
Cibc-5	England	*RPM1*	X
Hr-10	England	*RPM1*	√
Hr-5	England	*RPM1*	√
Nfa-10	England	*RPM1*	√
Nfa-8	England	*RPM1*	X
Sq-1	England	*RPM1*	X
Sq-8	England	*RPM1*	X
Est-1	Estland	*RPM1*	√
Tamm-2	Finland	*RPM1*	√
Tamm-27	Finland	*RPM1*	√
Gy-0	France	*RPM1*	√
Lz-0	France	*RPM1*	X
Ra-0	France	*RPM1*	X
Ren-1	France	*RPM1*	√
Ren-11	France	*RPM1*	√
Bay-0	Germany	*RPM1*	X
Ei-2	Germany	*RPM1*	√
Ga-0	Germany	*rpml-null* (deletion)	X
Got-22	Germany	*RPM1*	X
Got-7	Germany	*RPM1*	X
Gu-0	Germany	*RPM1*	√
Mrk-0	Germany	*rpml-null* (deletion)	X
Mz-0	Germany	*rpml-null* (deletion)	X
Nd-1	Germany	*rpml-null* (deletion)	X
Wt-5	Germany	*rpml-null* (deletion)	X
Kas-2	India	*rpml-null* (deletion)	X
Bur-0	Ireland	*RPM1*	√
Ct-1	Italy	*RPM1*	√
Tsu-1	Japan	*RPM1*	√
Kz-1	Kazakhstan	*RPM1*	X
Kz-9	Kazakhstan	*RPM1*	√
Mt-0	Libya	*rpml-null* (deletion)	X
Bil-5	N. Sweden	*RPM1*	X
Bil-7	N. Sweden	*RPM1*	X
Eden-1	N. Sweden	*rpml-null* (deletion)	X
Eden-2	N. Sweden	*RPM1*	X
Fab-2	N. Sweden	*RPM1*	X
Fab-4	N. Sweden	*RPM1*	X
L6v-1	N. Sweden	*RPM1*	√
L6v-5	N. Sweden	*RPM1*	X
Nok-3	Netherlands	*RPM1*	√
Oy-0	Norway	*RPM1*	√
Col-0	Poland	*RPM1*	√
Ler-1	Poland	*RPM1*	√
Wa-1	Poland	*RPM1*	X
C24(CO-l)	Portugal	*rpml-null* (deletion)	X
Fei-0	Portugal	*RPM1*	X
N13	Russia	*RPM1*	√
ÖMö2-1	S. Sweden	*RPM1*	√
ÖMö2-3	S. Sweden	*rpml-null* (deletion)	X
Spr-1-2	S. Sweden	*RPM1*	X
Spr-1-6	S. Sweden	*RPM1*	√
Ull-2-3	S. Sweden	*rpml-null* (deletion)	X
Ull-2-5	S. Sweden	*RPM1*	√
Var2-1	S. Sweden	*RPM1*	X
Var2-6	S. Sweden	*RPM1*	√
Edi-0	Scotland	*RPM1*	√
Ll-0	Spain	*RPM1*	√
Pro-0	Spain	*RPM1*	√
Se-0	Spain	*RPM1*	√
Ts-1	Spain	*RPM1*	√
Ts-5	Spain	*RPM1*	X
Wei-0	Switzerland	*rpml-null* (deletion)	X
Kondara	Tajikistan	*RPM1*	X
Shakdara	Tajikistan	*RPM1*	√
Sorbo	Tajikistan	*RPM1*	√
KNO-10	USA (Midwest, Indiana)	*rpml-null* (deletion)	X
Kno-18	USA (Midwest, Indiana)	*RPM1*	X
Rrs-10	USA (Midwest, Indiana)	*rpml-null* (deletion)	X
Rrs-7	USA (Midwest, Indiana)	*RPM1*	√
Pna-10	USA (Midwest, Michigan)	*rpml-null* (deletion)	X
Pna-17	USA (Midwest, Michigan)	*rpml-null* (deletion)	X
Rmx-A02	USA (Midwest, Michigan)	*rpml-null* (deletion)	X
Rmx-A180	USA (Midwest, Michigan)	*RPM1*	√
Ws-0	Ukraine	*RPM1*	√
Ws-2	Ukraine	*RPM1*	√
Yo-0	USA	*rpml-null* (deletion)	X

*Genotypes and phenotypes as defined in Table A1*.

**Table A3:** **Survey of sequence variation at *RPM1* in worldwide sample using diagnostic markers**.

	Position: CAPs 2 (Col-0 type)	CAPs 2	Large insertion
CAPs 1 (Col-0 type)	33 (1/33 susceptible)	1 (1/1 susceptible)	0
CAPs 1 (3 amino acid deletion)	56 (4/56 susceptible)	44 (44/44 susceptible)	5 (5/5 susceptible)

*Number of accessions containing marker combinations as described in Figure A1. The combination of CAPs marker 1 with either: (a) CAPs marker 2 or (b) a large insertion in the RPM1 gene, is associated with susceptibility in our sample of *A. thaliana* accessions containing the *RPM1* coding region*.
